# Microwave-Driven Nonoxidative and Selective Conversion of Methane to Ethylene over Mn-Based Catalysts

**DOI:** 10.1021/acs.iecr.5c02894

**Published:** 2025-11-11

**Authors:** Snehitha Reddy Baddam, Changle Jiang, Manohar Reddy Poreddy, Kshitij Tewari, Brandon Robinson, Yuxin Wang, Srinivas Palanki, Jianli Hu

**Affiliations:** Department of Chemical and Biomedical Engineering, 5631 West Virginia University, Morgantown, West Virginia 26505, United States

## Abstract

Recent advancements in microwave-driven nonoxidative catalytic synthesis of C_2_H_4_ from CH_4_ coupling offer a promising, energy-efficient, and eco-friendly alternative to conventional methods, where selective heating under microwave irradiation enables comparable conversions at substantially lower bulk temperatures and shorter reaction times. This study explores the performance of an MnO_X_-based catalyst supported on CeO_2_ and HY zeolite (silica-to-alumina ratio = 5.1) for the nonoxidative coupling of CH_4_ (NOCM) under microwave irradiation. Inspired by the well-established efficacy of MnO_X_ catalyst in oxidative CH_4_ coupling (OCM), their application in NOCM has also shown significant performance. The catalytic system achieved 15% CH_4_ conversion and 99% selectivity toward C_2_ hydrocarbons and maintained 64% selectivity toward C_2_H_4_ surpassing the yields reported in the literature even at higher temperatures (700–1000 °C). Catalyst performance was correlated with measurements by in situ Raman spectroscopy, and additional characterizations were performed using H_2_–temperature programmed reduction , NH_3_–temperature programmed desorption, and BET surface area analysis to understand structural changes during the reactions. These findings suggested that Mn functions as active sites for CH_4_ activation in nonoxidative environments while also promoting efficient C–C coupling under microwave irradiation.

## Graphical abstract

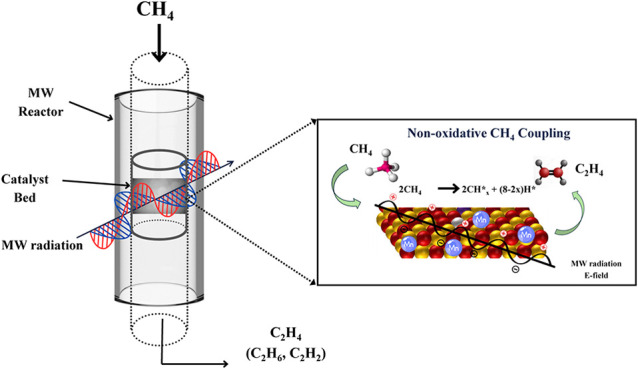

## 1 Introduction

Ethylene is a fundamental petrochemical feedstock utilized in the production of polymers, chemicals, and materials gripping the global chemical industry.^1^ The worldwide production capacity of C_2_H_4_ has increased from 214.3 million metric tons (MMT) in 2021 to 227.6 MMT in 2023 and is projected to reach around 287 MMT by 2030. Economically, the C_2_H_4_ market is expected to grow at a compound annual growth rate (CAGR) of ∼7%, reaching a valuation of approximately $161 billion by 2026.^2^ Conventionally, C_2_H_4_ is produced through steam cracking, a highly energy-intensive endothermic process that involves the pyrolysis of naphtha derived from a petroleum refinery.^2,3^ There are concerns over the depletion of non-renewable fossil resources. There is an urgent need to develop alternative, energy-efficient, and sustainable pathways for C_2_H_4_ production. Conventional technologies available to produce C_2_H_4_ include (i) steam cracking of hydrocarbon feedstocks, (ii) dehydrogenation and oxidative dehydrogenation of alkenes, and (iii) dehydration of ethanol.^3^ In these processes, hydrocarbon feedstocks are thermally converted via gas-phase radical reactions into C_2_H_4_ and other light olefins, as well as H_2_ coproducts, at elevated temperatures exceeding 750 °C. The high endothermicity of these cracking reactions, along with the complex downstream cryogenic separation steps, renders such processes both energy- and carbon-intensive, with an associated emission of approximately 1–2 tons of CO_2_ per ton of C_2_H_4_ produced, depending on the feedstock.^4^

While steam cracking remains the dominant commercial route owing to its ability to coproduce valuable olefins such as propylene and butadiene, it also yields undesired byproducts such as CH_4_ and fuel oil. Alternative routes like catalytic pyrolysis, ethanol dehydration, and membrane-assisted C_2_H_6_ dehydrogenation have been explored to enhance the efficiency and sustainability. Catalytic pyrolysis, for instance, targets higher C_2_H_4_ yields by using low surface-area alumina supports but is constrained by challenges such as coking, water–gas shift activity, and limited long-term olefin selectivity.^4–6^ To overcome these challenges, a range of alternative C_2_H_4_ production methods has emerged. Among the various routes explored for direct CH_4_ to C_2_H_4_ conversion, oxidative coupling of CH_4_ (OCM) and nonoxidative coupling of CH_4_ (NOCM) have received considerable attention. OCM and NOCM are among the most extensively used routes for C_2_H_4_ production. While OCM involves the reaction of CH_4_ with O_2_ to produce C_2_H_4_ and H_2_O, benefiting from favorable thermodynamics, it faces inherent challenges such as overoxidation, leading to undesirable CO and CO_2_ byproduct formation. In contrast, NOCM enables the formation of C_2_H_4_ and H_2_ in the absence of oxidants, effectively circumventing CO_2_ emissions.^7^ Despite requiring very high operating temperatures and facing issues related to coke formation and equilibrium limitations, NOCM offers a compelling advantage in terms of carbon efficiency and selectivity because it directly transforms CH_4_ into value-added C_2_ hydrocarbons, such as C_2_H_4_ and C_2_H_6_, without compromising the carbon balance of the system.^4,8–12^

In both OCM and NOCM routes, CH_4_ is activated over catalytic metallic sites to generate methyl radicals (*CH_3_), which desorb into the gas phase and undergo homogeneous coupling reactions to form C_2_ hydrocarbons. Initially, two *CH_3_ combine to form C_2_H_6_, which then dehydrogenates to C_2_H_4_ under high-temperature conditions (typically >1000 K). These radical-mediated pathways avoid external oxidants, reducing the risk of overoxidation and CO_2_ formation. Moreover, suppressing coke formation, which is a common challenge in hydrocarbon upgrading, is addressed by optimizing the catalyst structure to minimize surface C–C coupling and enhance gas-phase interactions.^13,14^ For instance, Sot et al., reported the formation of higher hydrocarbons (C_2_H_6_, C_2_H_4_, C_2_H_2_, and C_6_H_6_) with a total selectivity of ∼20% with only 3% CH_4_ conversion at 1080 °C.^14^ Guo et al., achieved up to 48% CH_4_ conversion and 48.4% selectivity toward C_2_H_4_ at 1090 °C, highlighting the feasibility of significant conversion under optimized conditions.^15^ Catalysts such as Mo_2_C/ZSM-5,^16^ Mo–Fe-/ZSM,^17^ and GaN/SBA15–5,^18^ have also been explored for NOCM, typically yielding 2% CH_4_ conversion below 1000 °C. While Pt-based catalysts have been extensively studied and shown favorable selectivity, their high cost and low CH_4_ conversion under NOCM conditions limit scalability.^19^ To overcome these thermal and efficiency limitations, alternative energy delivery methods such as microwave heating have gained attention over time.

The use of microwave reactors offers a promising strategy to enhance the efficiency of highly endothermic processes such as NOCM.^15^ Microwaves, which are electromagnetic radiation with wavelengths in the range of 1 mm to 1 m (300 GHz–300 MHz), can interact with dielectric materials to induce rapid and localized heating.^18^ In contrast to conventional heating methods based on conduction, convection, and radiation, microwave irradiation enables direct and volumetric energy transfer to the catalyst bed, either through intrinsic absorption by the catalyst or via a microwave-absorbing susceptor.^9^ This localized heating is particularly beneficial for CH_4_ conversion reactions, as metal species exhibit higher microwave absorption on catalyst surfaces due to their greater dielectric loss or electrical conductivity compared to inert supports like SiO_2_, Al_2_O_3_. As a result, microwave irradiation preferentially energizes the active metal sites, which enhances the reaction rates and product selectivity.^3,18^

Additionally, microwave heating mechanisms such as dipolar rotation and Debye relaxation induce energy transfer at the molecular level, where microwave energy is selectively delivered to the catalyst bed without significantly heating the surroundings.^20,21^ Hence, this selective heating at the interface between the active metal sites and reaction intermediates can improve the yield of products, promote the C–H bond activation, and limit coke formation, thereby making microwave-assisted NOCM a highly attractive strategy for direct conversion of CH_4_.^22^

This work reports, for the first time, the performance evaluation of Mn-based catalysts in methane coupling reactions under microwave irradiation. A novel investigation of such catalysts was carried out using in situ Raman spectroscopy. In addition, comprehensive characterizations were conducted toward the catalyst, such as XRD, BET, TGA, and NH_3_-TPD, helping reveal the mechanism of the CH_4_ coupling reaction. Notably, Keller^1^ demonstrated the superior performance of MnO_X_-based catalysts in OCM, highlighting their potential for CH_4_ activation. Building upon these findings, the present study investigated MnO_X_ individually supported on CeO_2_ and HY zeolite (silica to alumina ratio (SAR) = 5.1) for the NOCM under microwave irradiation. CeO_2_ was selected as a support for its redox flexibility and ability to generate oxygen vacancies, which promote CH_4_ activation. HY zeolite, on the other hand, was chosen for its high surface area and strong acidity, which enhance Mn dispersion and C_2_ selectivity. Furthermore, both CeO_2_ and HY are microwave-susceptible materials capable of efficient dielectric heating, which aids in localized energy adsorption and localized energy absorption and electron transfer during the reaction. The strategy aims to exploit the redox activity of Mn species, the O_2_ vacancy formation and chemisorption capacity of CeO_2_, and the high surface area of HY zeolite for enhancing CH_4_ activation and C_2_H_4_ selectivity. By integrating these tailored catalytic properties with microwave heating, this work establishes a selective and efficient pathway for CH_4_ conversion to value-added C_2_ hydrocarbons.

## 2 Experimental Section

### 2.1 Chemicals

Manganese­(II) nitrate tetrahydrate [Mn (NO_3_)_2_·4H_2_O,98%], was purchased from Thermo Scientific and used as a metal precursor. Cerium­(IV) oxide (CeO_2_) support was procured from Sigma-Aldrich. The HY Zeolite support, with SiO_2_/Al_2_O_3_ molar ratio (SAR) of 5.1, was supplied by Zeolyst International.

### 2.2 Catalyst Preparation

Both catalysts were synthesized via the incipient wetness impregnation method, following the procedure discussed by Keller et al., using a metal loading of 5 wt % Mn. The required amount of manganese­(II) nitrate tetrahydrate [Mn (NO_3_)_2_·4H_2_O,98%] was dissolved in distilled water and impregnated onto the cerium­(IV) oxide (CeO_2_) and HY zeolite (SAR = 5.1) supports. The impregnated samples were first dried at 60 °C for 30 min, followed by further drying at 110 °C for 2–3 h. The dried materials were then calcined in air at 550 °C for 4 h to obtain the active catalysts. Prior to impregnation, the NH_4_^+^-form HY zeolite was converted to its protonated form by calcination in air at 550 °C for 4 h in a muffle furnace.

### 2.3 Catalytic Reaction Process

The performance of the prepared catalysts was evaluated in a 3 kW, 2.45 GHz magnetron microwave reactor equipped with a short-wave infrared (SWIR) system. The experiments were conducted with a 2.45 GHz Sairem 3 kW magnetron microwave interfaced with an automatic 4 stub-impedance tuner, sliding short circuit, and a Monomode high-temperature cavity operating in the TE_10_ mode. The maximum allowable power applied during the experiments was 300 W. To help enhance temperature uniformity, an aluminum insulation package was employed to minimize heat loss and maintain stable operating conditions. Temperature control was managed via PID feedback integrated into the reactor software. The pyrometers were factory-calibrated, and the microwave waveguide was carefully tuned to minimize reflected microwave power, which was maintained as close to zero as possible throughout all experimental runs. The catalyst bed was prepared by loading the catalyst into a quartz tube, with a packed bed height of approximately 1.5–2.0 cm. The catalyst bed was positioned upright to ensure optimal focusing of microwave radiation, as shown in Figure 1. A quartz rod was inserted above the bed to prevent catalyst movement during gas flow. For experiments using CeO_2_-supported catalysts, ∼1.5 g of catalyst was loaded, whereas only ∼ 0.5 g was used for HY-supported catalysts due to their significantly higher BET surface area. Prior to each reaction, the catalyst bed was flushed with N_2_ with 30 sccm flow rate to purge the system and provide a stable baseline. During the reaction, the feed flow was set to a total of 30 sccm with a CH_4_:N_2_ ratio of 80:20 i.e., 24 sccm CH_4_ and 6 sccm N_2_. The reactions were investigated at 650, 700, and 750 °C temperatures. To further examine the effect of flow rate on CH_4_ conversion and product selectivity, additional experiments were conducted with 25% and 50% increases in total flow rate.

**Figure 1:**
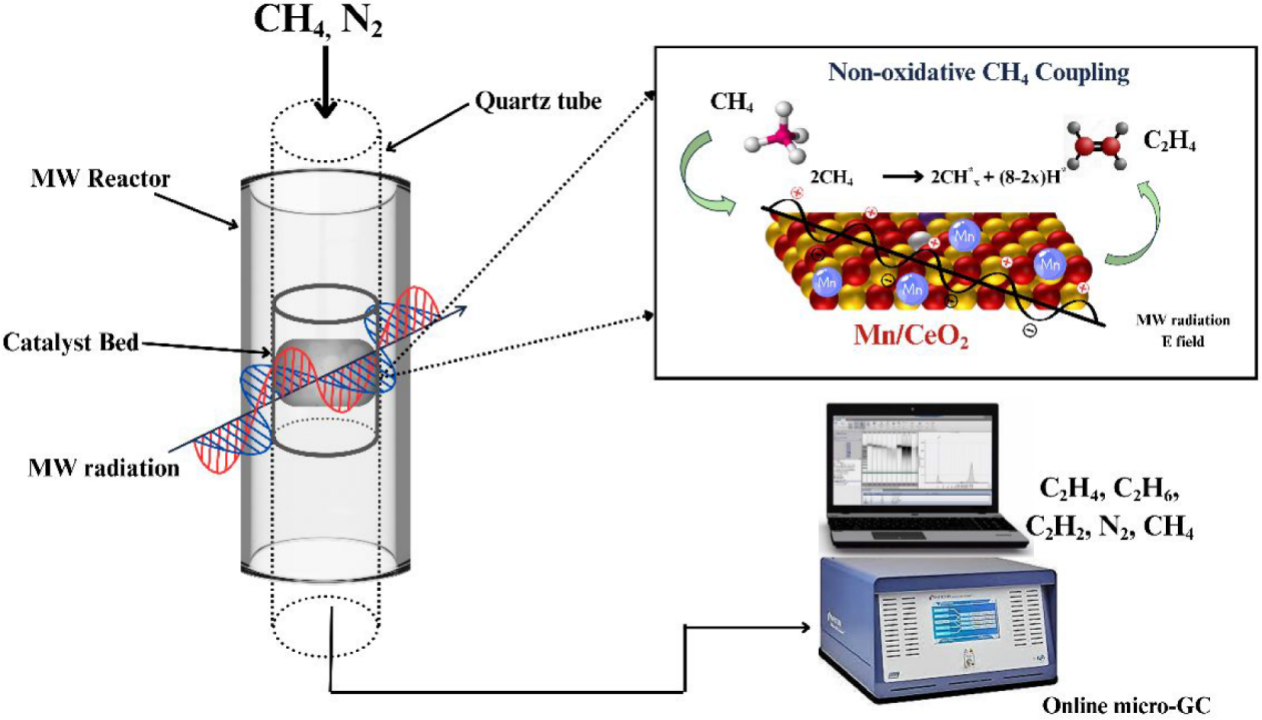
Schematic illustration of the microwave reactor system and the proposed reaction mechanism for MnO_X_/CeO_2_ catalytic system for nonoxidative methane coupling.

Product gas analysis was performed using a two-column Micro Gas Chromatography (Micro-GC) system (Agilent Technologies). Nitrogen was used as an internal standard for all calculations of conversion and selectivity and also served as the balance gas owing to its chemical inertness under the reaction conditions, with no nitrogen containing byproducts such as HCN detected in the effluent stream. The following equations (i–iii) were used:

i. CH_4_ conversion:

$$C_{\mathrm{Methane}}=\frac{\mathrm{Methane}\;\mathrm{Input}-\mathrm{Methane}\;\mathrm{Output}}{\mathrm{Methane}\;\mathrm{Input}}\times 100$$

ii. Selectivity of C_2_H_4_ products:

$$S_{\mathrm{Ethylene}}=\frac{\mathrm{Product}\;(\mathrm{mol}\%)\;\times \;\mathrm{Product}\;\mathrm{carbon}}{\sum \mathrm{Product}\;(\mathrm{mol}\%)\;\times \;\mathrm{Product}\;\mathrm{carbon}}\times 100\%$$

iii. Yield of C_2_H_4_:

$$Y_{\mathrm{Ethylene}}=S_{\mathrm{Ethylene}\;}\times \;CH_{4\;}\mathrm{conversion}\;\times \;100\%$$

MethaneOutput=Totalfloerate×MethaneConcentration(fromGC)

$$\mathrm{Total}\;\mathrm{flowrate}=\frac{\mathrm{Input}\;\mathrm{flowrate}\;\mathrm{of}\;\mathrm{Nitrogen}}{\mathrm{Nitrogen}\;\mathrm{Concentration}\;\left(\mathrm{from}\;GC\right)}$$

where *C*_*Methane*_ = Conversion of CH_4_, *S*_*Ethylene*_ = Selectivity of C_2_H_4_, *Y*_*Ethylene*_ = Yield of C_2_H_4_

### 2.4 Catalyst Characterization

Powder X-ray diffraction (PXRD) was carried out using a PANalytical X’Pert Pro diffractometer equipped with Cu Kα radiation, operating at 45 kV and 40 mA. The diffraction patterns were recorded over a 2θ range of 5° to 110° using an X’celerator solid-state detector, with a scan rate of 4.8° per minute. This analysis was used to confirm the crystallinity of the catalyst framework and identify any structural changes postmetal loading.

Hydrogen temperature-programmed reduction (H_2_-TPR) was carried out using a Micromeritics Autochem 2950 system to investigate the redox behavior of the catalysts. Approximately 200 mg of catalyst was pretreated in a Helium flow (50 mL/min) at 150 °C for 1 h, followed by cooling to 100 °C. The gas flow was then switched from He to 10%H_2_/Ar, and the system was allowed to stabilize for 20 min. The reduction profile was then recorded by heating the sample from 100 to 900 °C at a rate of 10 °C/min under a 10% H_2_/He gas mixture flowing at 50 mL/min.

NH_3_-temperature-programmed desorption (NH_3_-TPD) was performed using a Micromeritics AutoChem 2950 system to investigate the surface acidity of the catalysts. Approximately 200 mg of catalyst was first pretreated under He flow (50 mL/min) at 250 °C for 5 min, followed by cooling to 100 °C. After allowing the temperature to stabilize for 10 min, the gas flow was switched to a 15% NH_3_/He mixture, and the sample was exposed to this flow for 30 min to ensure thorough adsorption. The system was then held until a stable baseline was achieved. The TPD profile was subsequently recorded by heating the sample from 100 to 750 °C at a rate of 10 °C/min under a continuous flow of 15% NH_3_/He (50 mL/min).

N_2_-physisorption measurements were performed using a Micromeritics ASAP 2020 Plus instrument to evaluate the textural properties of the catalysts along with its supports. Prior to analysis, approximately 1 g of CeO_2_ support was degassed under vacuum at 300 °C for 1h, while 200 mg of the HY zeolite support was degassed at 300 °C for 4 h to eliminate moisture and surface impurities, ensuring clean surfaces for accurate adsorption measurement. This pretreatment ensured that the sample surfaces were clean and free of contaminants that could interfere with accurate adsorption measurements. Adsorption–desorption isotherms were recorded at −196 °C using high-purity nitrogen gas. The specific surface area was calculated using the Brunauer–Emmett–Teller (BET) method, and micropore volume and external surface area were determined from the t-plot method. The pore size distribution was calculated from the desorption branch of the isotherms.

Thermogravimetric analysis was conducted using a STD-Q650 instrument (TA Instruments, USA). The samples were initially heated from room temperature to 100 °C at a rate of 10 °C/min and held isothermally for 30 min to remove physiosorbed moisture. Subsequently, the temperature was increased from 100 °C to 900 °C at the same heating rate under a continuous flow of air, followed by an isothermal hold at 900 °C to evaluate thermal stability and decomposition behavior.

In-situ Raman spectroscopy was used to investigate the evolution of Mn species under temperature- programmed and simulated NOCM conditions. The experiments were performed using a Renishaw InVia Raman spectrometer equipped with a 532 nm green laser as the excitation source. In-situ measurements were carried out at 700 °C under a continuous CH_4_ and N_2_ flow using a specialized high-temperature Raman cell. The temperature was increased from room temperature to 700 °C at a controlled heating rate of 25 °C per minute, allowing real-time observation of structural changes in the catalyst. The Raman shift was calibrated using the silicon reference peak at 520.7 cm^–1^ to ensure spectral accuracy.

## 3 Results and Discussion

### 3.1 Physicochemical Properties of the Prepared Catalysts

#### 3.1.1 XRD of the Catalysts

Figure 2a presents the XRD patterns of blank CeO_2_, fresh MnO_X_/CeO_2_, and spent MnO_X_/CeO_2_ catalysts. The prominent diffraction peaks observed at 2θ = 28.5°, 33.1°, 47.4°, and 56.3° are attributed to the (1 1 1), (0 0 2), (0 2 2), and (1 1 3) crystal planes of CeO_2_, consistent with the fluorite-type cubic phase (JCPDS 98–002–8753, space group F_m3m_).^23,24^ These sharp and intense peaks indicate high crystallinity of the CeO_2_ support. Upon Mn doping, no additional diffraction peaks corresponding to MnO_X_ species were detected, suggesting either a high dispersion of MnO_X_ on the CeO_2_ surface or the successful incorporation of Mn ions into the CeO_2_ lattice.^24^ The slight broadening of diffraction peaks and decrease in intensity in the fresh and spent MnO_X_/CeO_2_ samples suggest a reduction in crystallite size and a partial loss of crystallinity, likely due to lattice distortion induced by Mn incorporation. Additionally, a very low-intensity peak around 26.9° in the spent catalyst corresponds to disordered carbon, indicating limited coke formation.

**Figure 2:**
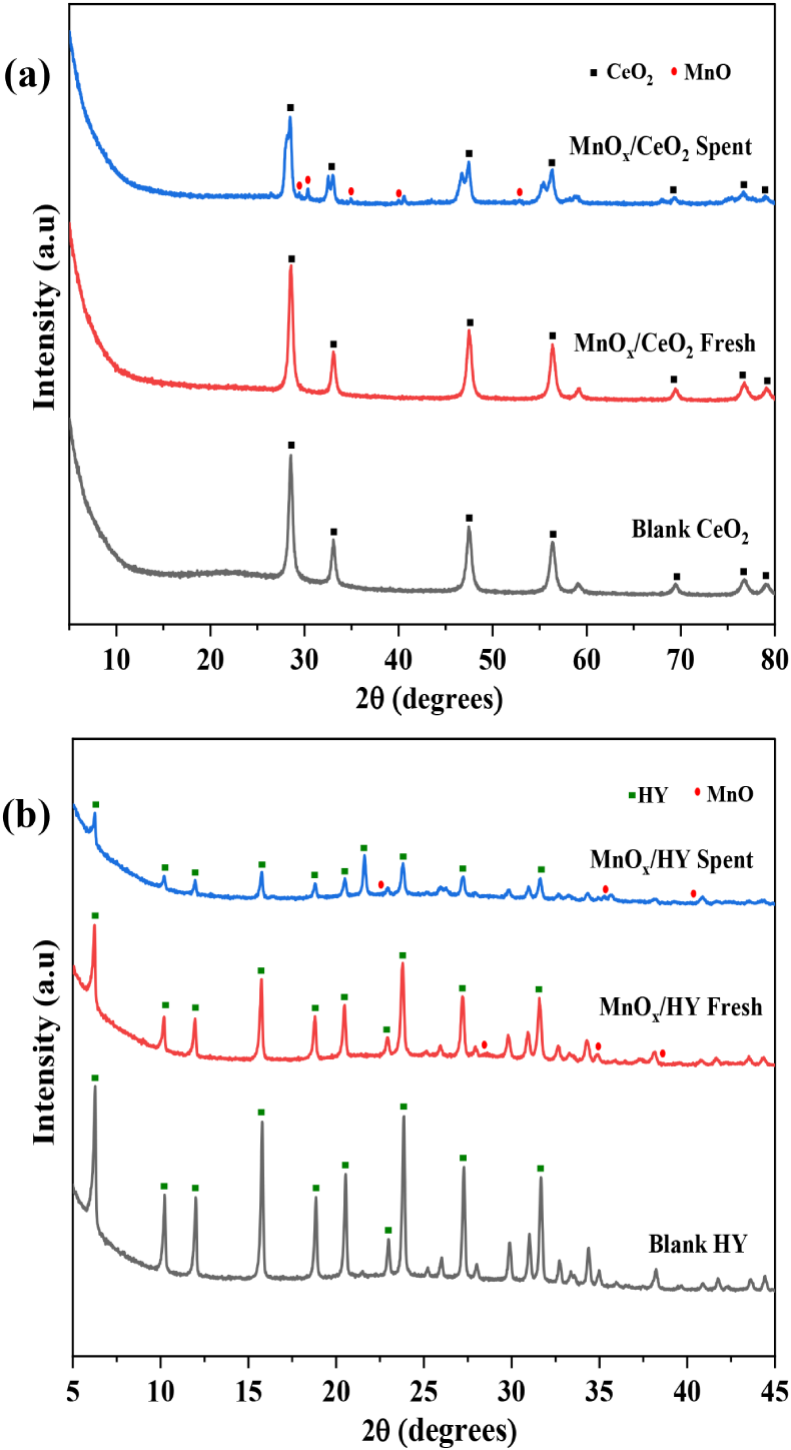
XRD pattern of blank supports, fresh and spent catalysts of (a) MnO_X_/CeO_2_ and (b) MnO_X_/HY.

Figure 2b shows the XRD patterns of blank HY zeolite, fresh MnO_X_/HY, and spent MnO_X_/HY catalysts. The blank HY sample exhibits characteristic peaks at 2θ = 6.29°, 10.28°, and 15.87°, corresponding to the (1 1 1), (0 2 2), and (1 3 3) planes (JCPDS 98–002–4869), confirming the typical crystalline structure of HY zeolite.^25^ The Mn-doped HY sample retains most of the structural features, with minor shifts observed at 14.1°, 15.83°, and 23.9°, corresponding to the (1 1 1), (1 3 3), and (3 3 5) planes (JCPDS 98–003–6206), indicating that the structural integrity of the zeolite is largely preserved upon Mn incorporation. However, the spent MnO_X_/HY catalyst exhibits notable changes in the diffraction pattern, with additional peaks appearing at 5.74°, 10.41°, 25.0°, 25.4°, 28.1°, 28.8°, 31.1°, 35.7°, 36.5°, and 40.6°, corresponding to various planes (1 0 2), (1 0 1), (2 1 0), (2 0 3), (3 0 1), (3 0 2), (0 2 0), (1 0 4), (2 2 1),(2 2 3) identified with JCPDS 98–003–0870. These changes indicate the formation of new crystalline phases, partial dealumination of the zeolite, or the accumulation of MnO_X_ phases and carbonaceous residues during the reaction.

#### 3.1.2 H_2_-TPR Analysis of MnO_X_ Catalysts Supported on CeO_2_ and HY: Influence of Support on Redox Behavior

Figure 3 shows the H_2_-TPR profiles of the blank supports and their Mn-loaded counterparts. The H_2_-TPR profile of pure CeO_2_ is characterized by three distinct reduction peaks. Two prominent peaks appear in the range of 400–600 °C, corresponding to the reduction of surface oxygen species.^24^ The first peak, observed near 400 °C, is attributed to the reduction of stoichiometric Ce^4+^–O-Ce^4+^ surface sites. The second peak, centered around 510 °C, is associated with the reduction of nonstoichiometric oxygen species, specifically Ce^3+^–O–Ce^4+^ surface linkages, which represent loosely bound surface-capping oxygen.^26^ These features highlight the redox flexibility of ceria, which readily transitions between Ce^4+^ and Ce^3+^ oxidation states,^24^ enabling its well-known oxygen storage and release capacity. A third, broader reduction peak emerges above 800 °C, corresponding to the reduction of bulk CeO_2_, indicating deeper lattice oxygen involvement in the redox process.^26^

**Figure 3:**
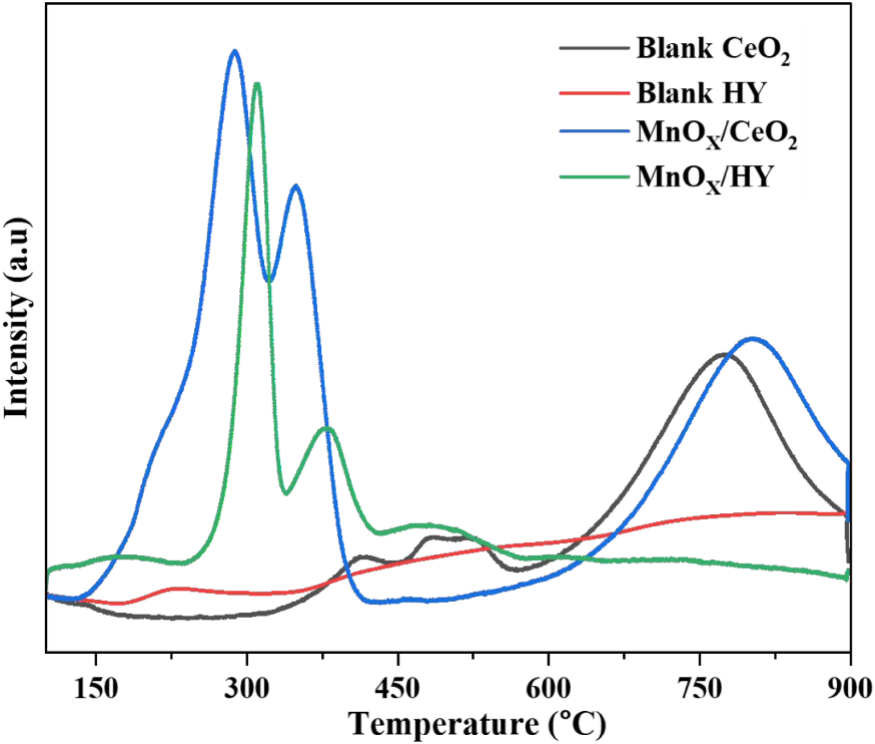
H_2_ -TPR profiles of blank CeO_2_ and HY supports and MnO_X_/CeO_2_ and MnO_X_/HY catalysts.

Upon Mn incorporation, a substantial modification of the CeO_2_ reduction behavior is observed. The H_2_-TPR profile of MnO_X_/CeO_2_ exhibits a notable shift toward lower reduction temperatures, indicating enhanced reducibility resulting from strong interactions between Mn species and the CeO_2_ support. Distinct new reduction peaks emerge around 290 and 350 °C, corresponding to the reduction of amorphous MnO_2_ and Mn_2_O_3_, respectively, along with the simultaneous formation of oxygen vacancies in the CeO_2_ lattice.^27,28^ Concurrently, the diminished intensity of original surface-related CeO_2_ peaks suggests partial substitution or modification of surface oxygen environments by Mn species. This behavior underscores the improved low-temperature redox capability of the Mn-loaded catalyst.

In contrast, the blank HY support exhibited a nearly featureless H_2_-TPR profile, consistent with its nonreducible aluminosilicate framework. However, after Mn impregnation, two new peaks emerged at approximately 310 and 390 °C, indicating the formation of Mn species that are weakly interacting with the HY surface but still contribute to hydrogen uptake at moderate temperatures. These results show that metal incorporation into both CeO_2_ and HY significantly enhances the redox behavior of the system.^29^

#### 3.1.3 NH_3_-TPD Analysis of Mn-Based Catalysts: Influence of Support on Surface Acidity

The surface acidity of the catalysts was examined using NH_3_-temperature-programmed desorption (NH_3_-TPD) as shown in Figure 4. The position and area of the desorption peaks reflect the strength and density of acid sites, respectively.^30^

**Figure 4:**
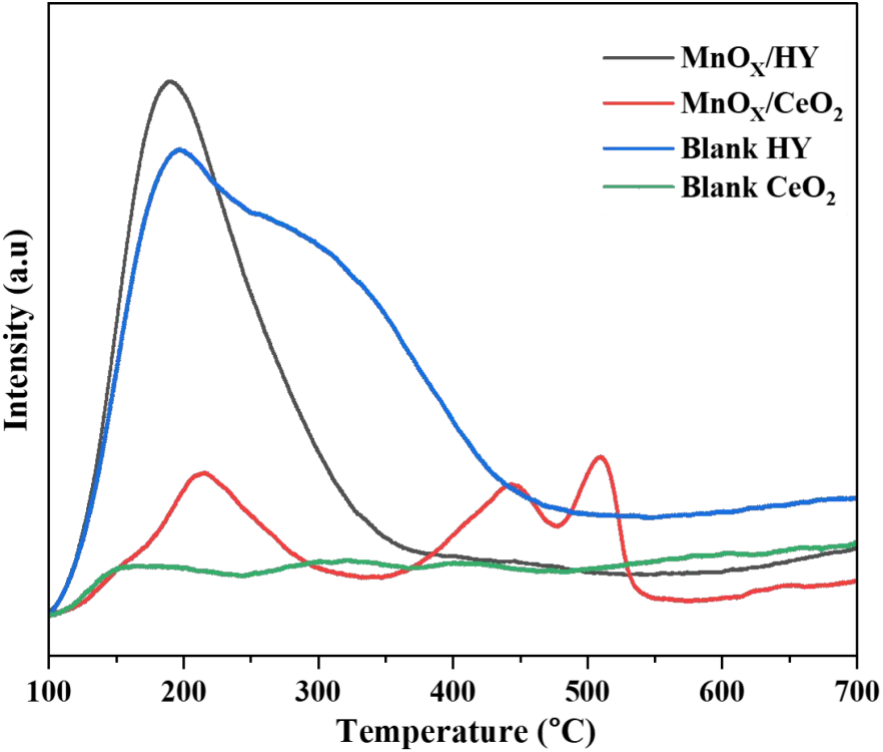
NH_3_-TPD profiles of blank CeO_2_ and HY supports and MnO_X_/CeO_2_ and MnO_X_/HY catalysts.

The NH_3_-TPD profile of HY (SAR = 5.1) zeolite displayed a major desorption peak around 250 °C, with a shoulder near 400 °C.^31^ The low-temperature peak corresponds to weak Lewis and/or Brønsted acid sites, while the shoulder at higher temperature indicates strong Brønsted acidity.

In contrast, Mn-doped CeO_2_ showed three distinct desorption peaks assigned to weak (100–250 °C), medium (350–450 °C), and strong (>450 °C) acid sites,^32^ indicating the presence of both Lewis and Brønsted acidic functionalities. The midrange peak (100–300 °C) is attributed to NH_3_ desorption from Lewis acid sites, whereas high-temperature peaks (400–550 °C) correspond to Brønsted acid sites.^33^ Blank CeO_2_ exhibited negligible NH_3_ desorption, confirming its inherently weak Lewis acidity. Although the total number of acid sites on MnO_X_/CeO_2_ (represented by the area under the peaks) is less than MnO_X_/HY catalyst, the strength of acid sites (represented by the temperature of the peaks) is much higher.

#### 3.1.4 BET Surface Area and Pore Size Analysis of Mn-Based Catalysts

The nitrogen adsorption–desorption analysis revealed notable differences in BET surface area and porosity between the CeO_2_ and HY supported catalysts. As shown in Table 1, the BET surface area of the blank CeO_2_ support was relatively low at 25.19 m^2^/g, which is consistent with previously reported values for pure Ceria, typically ranging from 20 to 65 m^2^/g depending on its synthesis conditions and morphology.^28^ Upon incorporation of 5 wt % Mn, the surface area further decreased to 15.23 m^2^/g, indicating possible pore blockage or surface coverage by MnO_X_ species during the impregnation process.^34^ In contrast, the HY zeolite support has shown a high BET surface area of 606.72 m^2^/g, which has decreased to 491.23 m2/g, possibly due to the pore blockage attributed to the loading of Mn metal to the zeolite channel.^27,35^ Additional information regarding the isotherm and pore size distribution are provided in the Figures S1 and S2.

**Table 1:** BET Surface Area of Blank Supports and Mn-Doped Supports

**Sample**	***S***_**BET**_ ^a^ **/** **(m** ^**2**^ **/g)**	***V***_**t**_ ^b^ **/** **(cm** ^**3**^ **/g)**	***D*** ^c^ **/(**nm)	***V***_**Micro**_ ^d^ **/** **(cm** ^**3**^ **/g)**
Blank CeO_2_	25.1947	0.014638	2.3240	0.000209
Blank HY (SAR = 5.1)	606.7187	0.340767	2.2466	0.300695
5%Mn/CeO_2_	15.2319	0.008859	2.3265	0.000459
5%Mn/HY	491.2250	0.275434	2.2428	0.242152

a BET (Brunauer–Emmett–Teller) specific surface area.

b Total pore volume, measured at *P*/*P* ^0^= 0.4304.

c Average pore diameter of samples, calculated as 4 *V*_t_/*S*_BET_.

d Micropore volume derived from t-plot.

#### 3.1.5 Thermogravimetric Analysis (TGA)

The thermogravimetric profiles of CeO_2_ and HY-supported catalysts are presented in Figure 5. The TGA experiments were conducted in air, and the observed weight loss was attributed to structural changes and oxygen release/uptake processes. The blank CeO_2_ support and fresh MnO_*x*_/CeO_2_ catalyst, as in Figure 5a, exhibited an initial weight loss below 200 °C, primarily associated with the desorption of physically adsorbed water and surface moisture. However, the fresh MnO_X_/CeO_2_ catalyst has shown two major weight loss events in the temperature range 550–650 °C and 650–800 °C, which can be attributed to the stepwise reduction of MnO_2_ to Mn_2_O_3_ and subsequently to MnO, where Mn reduces from Mn^4+^ → Mn^3+^ →Mn^2+^.^36^ These changes highlight the redox activity induced by Mn incorporation and the oxygen storage/release behavior promoted by the CeO_2_ support under elevated temperatures. The weight gain observed around 200 °C in the spent MnO_X_/CeO_2_ sample is attributed to reoxidation of reduced Mn species (Mn^0^ or MnO) and possible Mn carbides (MnC_X_) formed under reaction conditions, along with oxidation of surface carbon deposits. This low-temperature reoxidation step, commonly reported for Mn phases, indicates that Mn participates actively in redox cycling, complementing CeO_2_’s oxygen storage capacity.The reoxidation of the M-carbide and oxygen deficient Mn (due to the reductive atmosphere) is the reason for the uptake. However,the spent MnO_X_/CeO_2_ catalyst displayed weight loss of 0.6% in the 450–500 °C range, corresponding to the release of surface lattice oxygen, suggesting the retention of redox activity even after the reaction.^37^ This retention of redox functionality in the spent catalyst indicates that MnO_X_/CeO_2_ maintains its structural integrity and capacity for oxygen mobility even after prolonged reaction cycles. Such stability is crucial for sustained CH_4_ activation and C–C coupling under NOCM conditions, as the reversible redox transitions of Mn species (Mn^4+^ → Mn^3+^ → Mn^2+^) coupled with the oxygen storage and release properties of CeO_2_ ensure continuous regeneration of active sites. The observed weight loss pattern thus not only confirms the reducibility of Mn species but also demonstrates the strong metal–support interactions that contribute to the catalyst’s durability and performance under microwave irradiation.^36,37^

**Figure 5:**
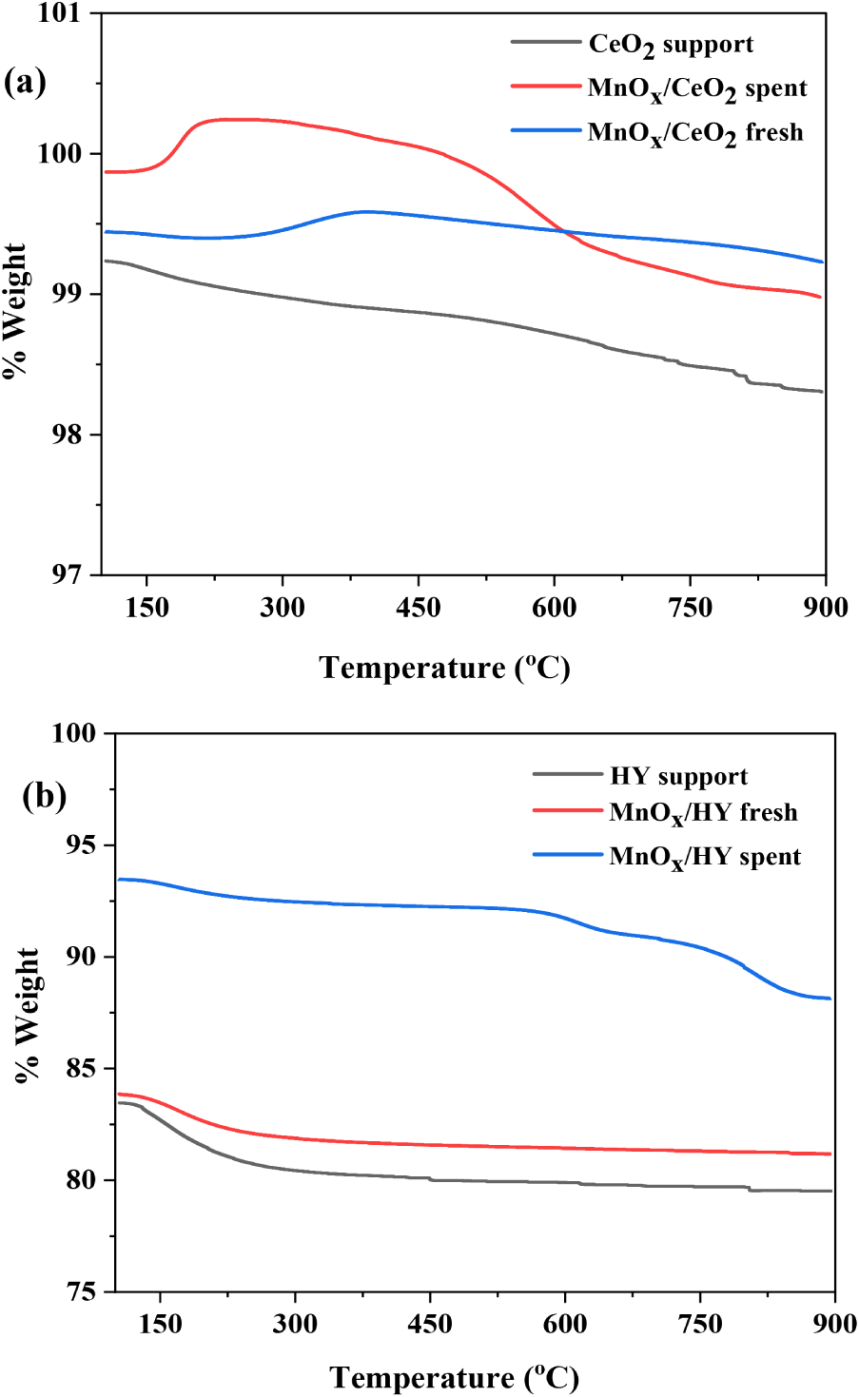
TGA of (a) blank CeO_2_ and MnO_X_/CeO_2_ fresh and spent catalysts, (b) blank HY support, and MnO_X_/HY fresh and spent catalysts.

In comparison, the TGA curves for the HY-supported systems, as in Figure 5b, indicate high thermal stability across all samples. The blank HY support and fresh MnO_*x*_/HY catalyst showed similar profiles, with minor weight losses of 3.96% and 2.12%, respectively, in the 100–300 °C region primarily due to dehydration.^18,38^ However, the weight loss in the spent catalysts occurred in three distinct steps: 1.74% at 600 °C, 0.89% at 695 °C, and approximately 2.69% at 900 °C. The spent MnO_*x*_/HY catalyst showed a noticeably higher total weight loss of 5.324% compared to the fresh samples, likely due to the removal of carbon or coke buildup accumulated during the reaction.^39^ Notably, the progressive weight loss above 600 °C in both the spent catalyst is attributed to the combustion of coke and possible deposition of residual impurities.^25^ These observations confirm that while some degree of carbon deposition occurs during the reaction, both MnO_X_/CeO_2_ and MnO_X_/HY catalysts exhibit excellent thermal stability and structural integrity postreaction. The DTA and DSC plots of MnOX/CeO2 and MnOX/HY are shown in the Figures S2–S4 in the Supporting Information.

#### 3.1.6 In-Situ Raman Spectroscopy of MnO_X_/CeO_2_

Ex-situ and in situ Raman spectroscopy were employed to investigate the evolution of Mn oxidation states and carbonaceous deposits during the (OCM) over MnO_X_/CeO_2_ catalysts. The ex-situ spectra (Figure 6a and b) provide information on structural and electronic changes after reaction, while the in situ spectra (Figure 6c and d) capture the dynamic transformations under CH_4_/N_2_ flow at elevated temperatures.

**Figure 6:**
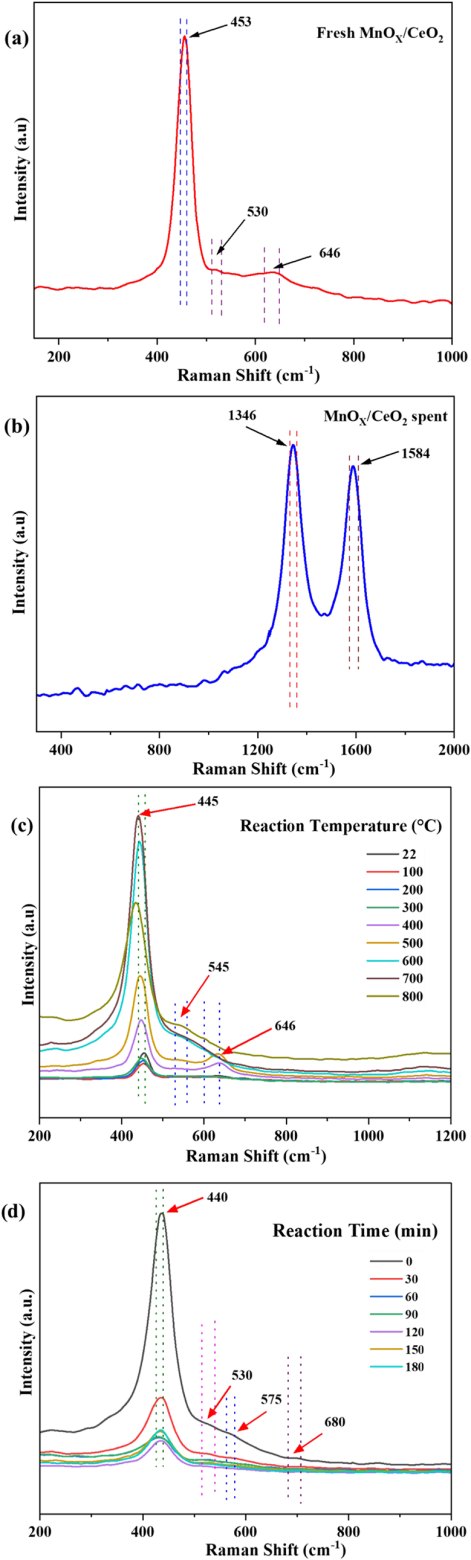
Ex-situ Raman measurement for (a) fresh MnO_X_/CeO_2_ (b) spent MnO_X_/CeO_2_ (c) In-situ Raman measurement for fresh MnO_X_/CeO_2_ as a function of temperature (d) as a function of reaction time at 700 °C.

The ex-situ Raman spectrum of the fresh MnO_X_/CeO_2_ catalyst exhibited a strong peak at 453 cm^–1^, corresponding to the F_2g_ Raman-active mode of CeO_2_. This mode reflects the symmetric breathing vibration of oxygen ions surrounding Ce^4+^ cations within the fluorite lattice structure.^24,40^ In contrast, the ex-situ spectrum of the spent catalyst taken from a sintered chunk of the catalyst bed revealed two distinct peaks at 1346 cm^–1^ and 1584 cm^–1^ which are the characteristics of disordered and crystalline carbonaceous species. The peak at 1584 cm^–1^ is associated with the in-plane stretching vibration of C = C stretching vibrational mode with E_2g_ symmetry found in well-ordered graphite structures and corresponds to the G band.^41^ The 1346 cm^–1^ peak, corresponding to the D-band, is attributed to zone-boundary A_1g_ phonons and arises from the vibrational modes of disordered graphitic domains. This mode becomes Raman active due to lattice symmetry breaking near the boundaries of microcrystalline carbon domains.^42,43^ This band corresponds to a decrease in symmetry near the boundaries of microcrystalline domains, where lattice distortions reduce the local symmetry from D_6h_ to C_3v_, or even to C_s_, enabling the D-band to appear in the Raman spectrum.^41^ Together, these features indicate the formation of amorphous and defective carbon structures on the catalyst surface during reaction.

In-situ Raman measurements conducted from room temperature to 800 °C under CH_4_/N_2_ flow showed notable transformations in both Mn and Ce species. As the temperature reaches 400 °C, a new peak appears at 646 cm^–1^, corresponding to MnO_2_ with Mn in the +4 oxidation state.^43^ This peak intensifies at 500 °C and subsequently shifts to 545 cm^–1^ by 800 °C, marking a significant change in Mn speciation. Initially, the ceria peak at 453 cm^–1^ appears to be dominant but its intensity decreases gradually over time. Simultaneously, the peaks associated with MnO at 530 cm^–1^, Mn_2_O_3_ at 545 cm^–1^, and MnO_2_ near 680 cm^–1^ are observed. As the reaction proceeds, signals corresponding to Mn_2_O_3_ and MnO_2_ diminish approximately after 30 min, while the MnO peak remains evident in the spent catalyst, which is also supported by H_2_-TPR analysis. This progression indicates the gradual reduction of Mn species from +4 and +3 oxidation states to the more stable +2 state under the reaction conditions.^43,44^

Additionally, a shift in the ceria Raman peak from 453 cm^–1^ to 440 cm^–1^ is observed during reaction. This red-shift can be attributed to the inhomogeneous strain broadening caused by variations in the particle size and lattice parameters within the crystalline domains. Such shifts are well explained by the phonon confinement model, which accounts for reduced phonon energies in nanostructured systems.^24,45^

### 3.2 Temperature Optimization for Non-Oxidative CH_4_ Coupling

To determine the optimum reaction temperature for the nonoxidative coupling of CH_4_ (NOCM) to C_2_H_4_, experiments were conducted at 650 °C, 700 °C, and 750 °C (Figure 7). Among these, 700 °C was found to be the most favorable temperature, offering a balance between selectivity, conversion, and thermal stability. At 650 °C, the system remained thermally stable but showed a lower C_2_H_4_ selectivity of approximately 52% (Figure 7) and a total C_2_ (C_2_H_4_ + C_2_H_6_ + C_2_H_2_) selectivity of around 94% (Figure 9), with CH_4_ conversion limited to ∼12% (Figure 8). When the temperature was raised to 700 °C, C_2_H_4_ selectivity improved significantly to about 65% as shown in Figure 7, and total C_2_ selectivity reached 99.5% as shown in Figure 9, with a modest increase in methane conversion to ∼ 15% (Figure 8). This temperature also maintained thermal stability over longer durations, making it optimal for continuous operation.^46,47^

**Figure 7:**
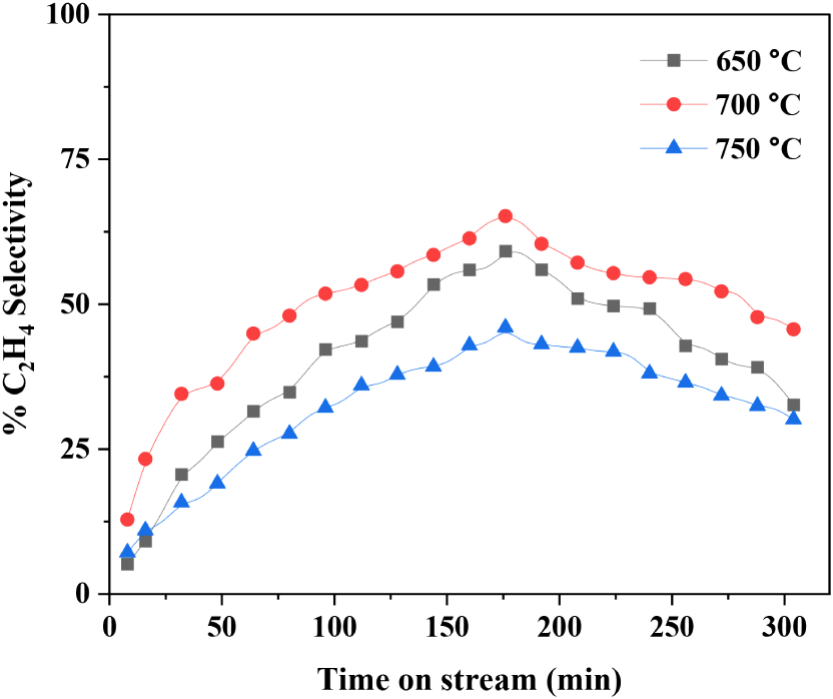
Optimization of reaction temperature to maximize C_2_H_4_ selectivity.

**Figure 8:**
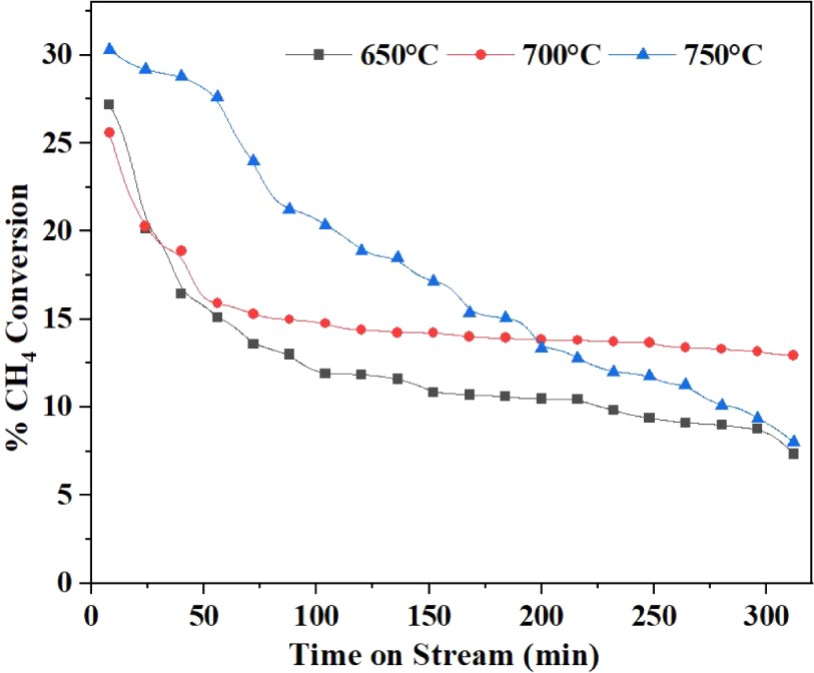
%CH_4_ conversion at various reaction temperatures.

**Figure 9:**
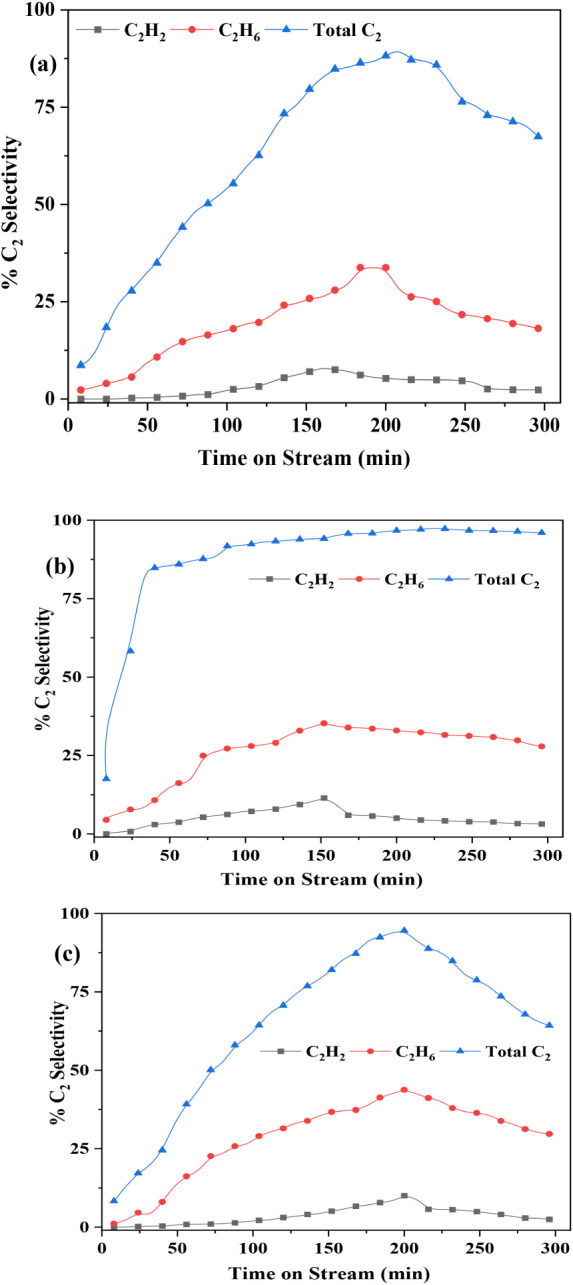
Selectivity of C_2_H_2_, C_2_H_4_, and total C_2_s at (a) 650 °C, (b) 700 °C, and (c) 750 °C.

In contrast, operation at 750 °C led to severe temperature instability, coking, and excessive formation of undesired byproducts such as C_2_H_2_ and aromatic compounds. Although CH_4_ conversion increased substantially to 27% at this higher temperature (Figure 8), the total C_2_ selectivity dropped sharply to ∼85% (Figure 9), compromising overall process efficiency. These results are consistent with findings reported by Julian et al.,^46^ and Desta et al.,^47^ who also identified 700 °C as the ideal operating point for maximizing ethylene yield in microwave-assisted NOCM systems.

#### 3.2.1 Catalytic Performance of MnO_X_/CeO_2_ And MnO_X_/HY at 700 °C

To evaluate the catalytic performance of the Mn-loaded supports for CH_4_ conversion and selective C_2_H_4_ production, both catalysts were tested under microwave irradiation, as shown in Figures 10 and 11. Figure 10 illustrates the CH_4_ conversion, and Figure 11 shows the %C_2_H_4_ selectivity and also the selectivity toward major hydrocarbon products (C_2_H_6_, C_2_H_4_, and C_2_H_2_) for 5 wt % MnO_X_ supported on CeO_2_ and HY zeolite. Both catalysts exhibited excellent selectivity toward C_2_H_4_ during the early phase of the reaction, known as the induction period, which was observed at approximately 50 min of time on stream (TOS). During this stage, the catalysts achieved their highest C_2_H_4_ production rates, highlighting a critical phase in which Mn predominantly exists in the +2-oxidation state, as confirmed by in situ Raman spectroscopy. The Raman spectra showed the emergence and stabilization of bands associated with Mn^2+^ species, indicating their active role in CH_4_ activation and C–C coupling. This behavior is attributed to the maximum microwave absorption capacity of the catalysts during this period, which generates localized hotspots that enhance catalytic activity. The correlation between the Mn oxidation state transition and catalytic performance, as captured by in situ Raman, strongly supports this interpretation.^18^

**Figure 10:**
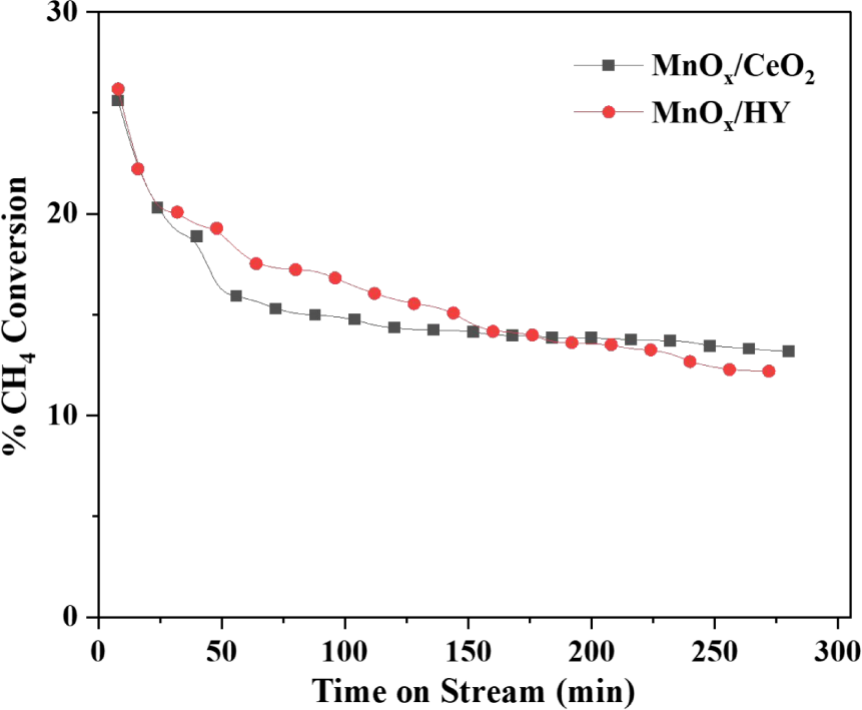
%CH_4_ conversion of 5 wt % MnO_X_/CeO_2_ and MnO_X_/HY at 700 °C.

**Figure 11:**
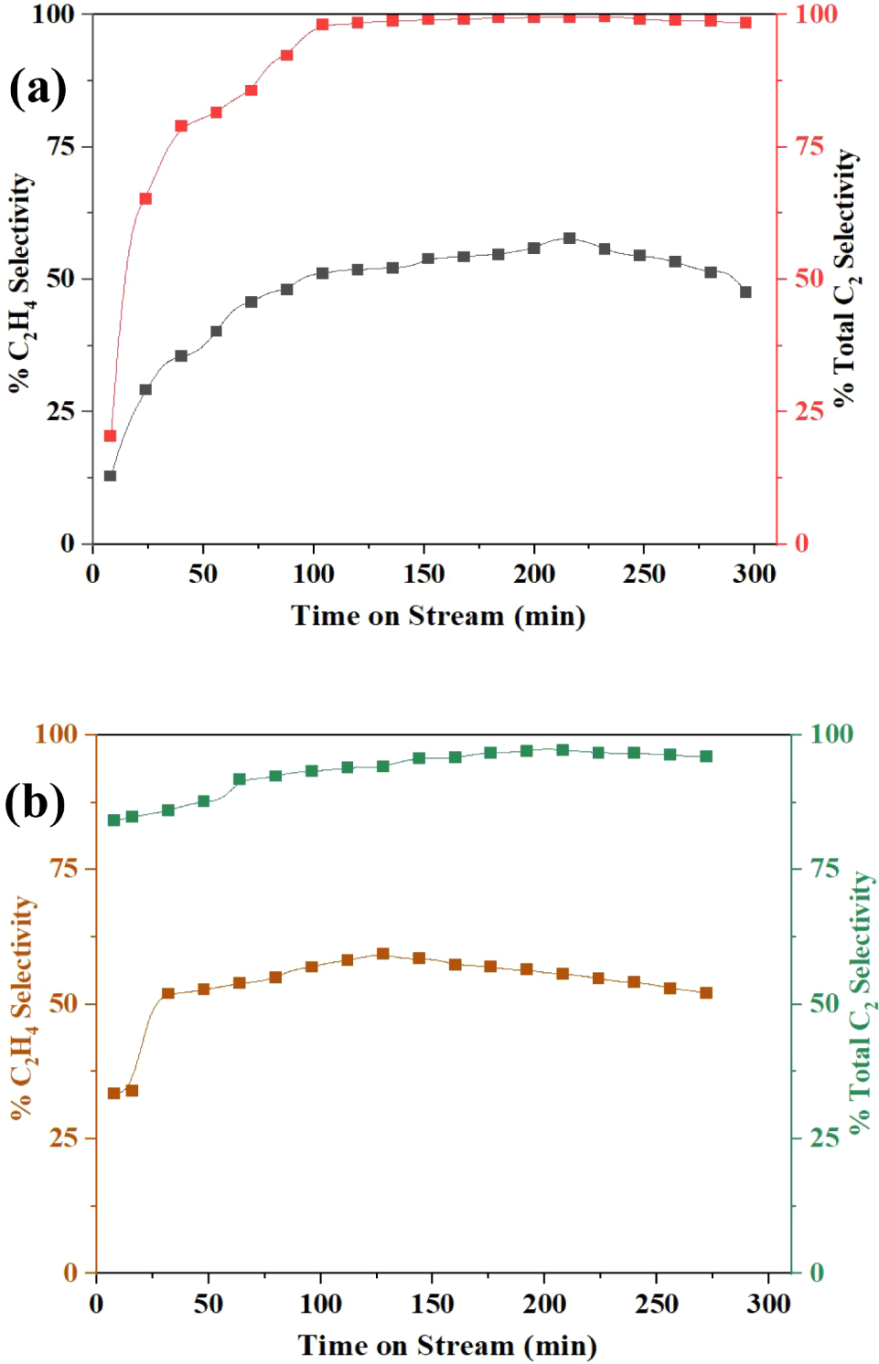
% C_2_H_4_ selectivity and total C_2_ selectivity using (a) MnO_X_/CeO_2_ and (b) MnO_X_/HY.

Throughout the reaction, the CH_4_ conversion remained relatively stable, ranging from 12% to 15% for both catalysts. Notably, both MnO_X_/CeO_2_ and MnO_X_/HY catalysts demonstrated C_2_H_4_ selectivity significantly higher than those reported in the literature^15,47^ for similar systems, underscoring the effectiveness of Mn species in promoting C–H bond activation and facilitating C–C coupling reactions. This superior performance can be correlated with the H_2_-TPR results, which revealed enhanced low-temperature reducibility and stronger Mn-support interactions, providing abundant redox-active sites that directly contribute to the observed improvement in C_2_ product selectivity. These dual functionalities of Mn, both in bond cleavage and in coupling pathways, appear to play a vital role in driving C_2_H_4_ production.

C_2_H_4_ emerged as the dominant product across both catalyst systems, with C_2_H_6_ also produced in appreciable quantities. Minor amounts of C_2_H_2_ and aromatic species (BTX: benzene, toluene, xylene) were detected, although their selectivity remained low. Importantly, the overall C_2_^+^ hydrocarbon selectivity exceeded 95% for both catalysts. Between the two systems, MnO_X_/CeO_2_ exhibited slightly superior performance, achieving higher C_2_H_4_ selectivity and total C_2_^+^ selectivity compared to the HY-supported catalyst. This difference may be attributed to the stronger metal–support interactions in the CeO_2_ system, together with the higher oxygen mobility of the CeO_2_ lattice, which facilitate C–H bond activation and C–C coupling, thereby enhancing C_2_ product yields, as also evidenced by the H_2_-TPR results.

#### 3.2.2 Catalytic Performance of MnO_X_/CeO_2_ and MnO_X_/HY at 700 °C under Varied Gas Hourly Space Velocity

To evaluate the effect of total flow rate on CH_4_ conversion and product selectivity, a series of experiments were conducted at three different CH_4_:N_2_ flow conditions using both MnO_X_/CeO_2_ and MnO_X_/HY (SAR = 5.1) catalysts. Figures 12 and 13 represent the results, showing CH_4_ conversion and total C_2_ hydrocarbon selectivity (C_2_H_6_, C_2_H_4_, and C_2_H_2_) as a function of time on stream (TOS). Additionally, CH_4_ conversion data under varied flow rates is presented in S5.

**Figure 12:**
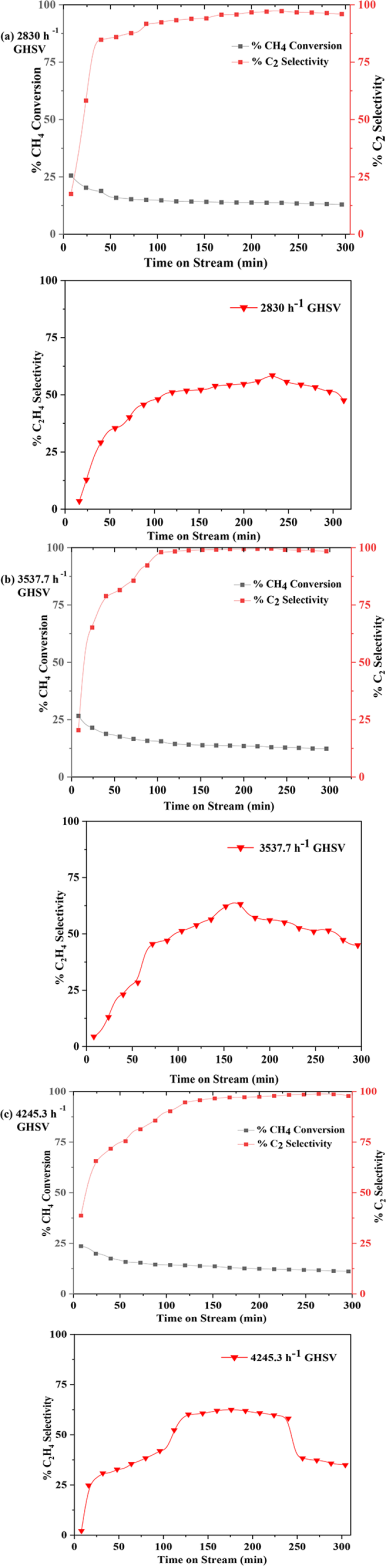
Influence of gas hourly space velocity (GHSV) on the performance of MnO_X_/CeO_2_ catalyst at 700 °C.

**Figure 13:**
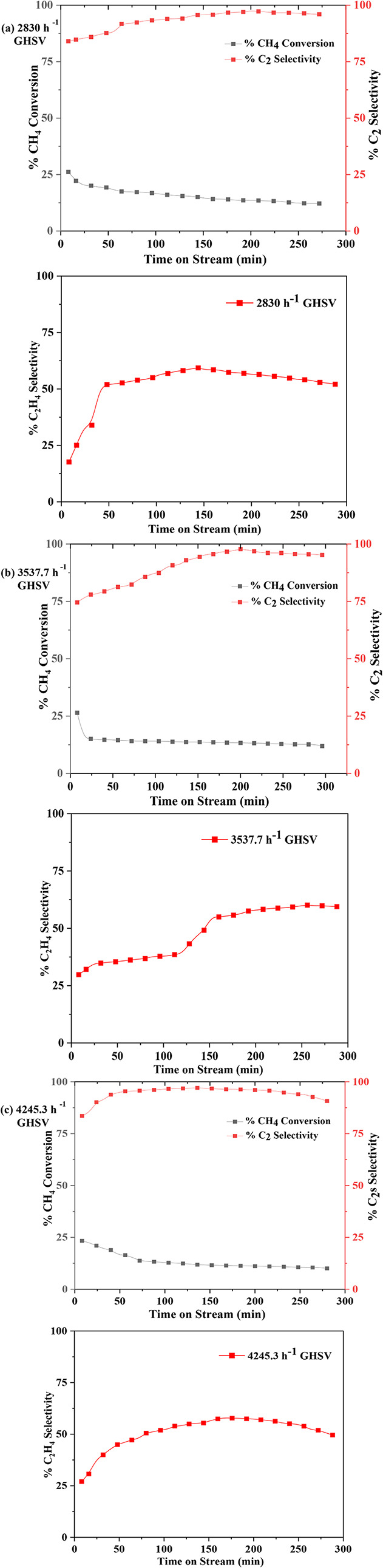
Influence of gas hourly space velocity (GHSV) on the performance of MnO_X_/HY catalyst at 700 °C.

For the MnO_X_/CeO_2_ catalyst, under the baseline flow rate of 24 mL/min CH_4_ and 6 mL/min N_2_, having a total flow of 2830 h^–1^ GHSV as in Figure 12, CH_4_ conversion initially reached ∼24% and gradually declined to stabilize around 15%. Concurrently, total C_2_ selectivity increased, peaking at nearly 92% before stabilizing at approximately 90%, with C_2_H_4_ selectivity reaching a maximum of 55%. Increasing the total flow rate by 25% to 3537.7 h^–1^ GHSV maintained CH_4_ conversion at comparable levels while significantly improving product selectivity where total C_2_ selectivity rising to 99.6%, and C_2_H_4_ selectivity peaked at 65%. At an even higher flow rate of 4245.3 h^–1^ GHSV (50% increase), CH_4_ conversion remained stable, C_2_H_4_ selectivity reached 62%, and total C_2_ selectivity initially peaked at 98%, followed by a slight decline to 90%. These trends indicate that higher flow rates favor the formation of C_2_H_4_ and C_2_H_6_, while suppressing C_2_H_2_ formation, likely due to reduced secondary reactions.

Similar behavior was observed for the MnO_X_/HY catalyst as shown in Figure 13. At a baseline flow rate of 30 mL/min, having a total flow of 2830 h^–1^, GHSV, CH_4_ conversion initially reached ∼26%, then gradually declined to ∼12% over time. During this period, total C_2_ selectivity remained high (∼95%), with C_2_H_4_ selectivity around 57%. Increasing the total flow rate by 25% led to further enhancement of C_2_H_4_ selectivity (∼61%) and peak total C_2_ selectivity of ∼99%, again reflecting a shift toward olefin production with reduced C_2_H_2_ generation. However, a further 50% increase in flow rate to 4245.3 h^–1^ GHSV resulted in a decrease in both total C_2_ and C_2_H_4_ selectivity, likely due to reduced residence time, limiting effective CH_4_ conversion and optimal product distribution.

The observed trends suggest that increasing flow rate helps minimize side reactions and promotes the desired C_2_ products by reducing the extent of secondary hydrogenation or overcracking reactions. Higher flow rates also reduce the opportunity for C_2_H_4_ to further react toward heavier or undesired products.^46,47^ On the other hand, excessively high flow rates may lower effective residence time and catalyst-contact efficiency, thereby limiting CH_4_ conversion. The superior performance of the Mn-promoted catalysts across these experiments can be attributed to their enhanced redox properties and surface acidity, as supported by H_2_-TPR and NH_3_-TPD results.

Figure 14a and b illustrate the detailed product distributions for MnO_X_/CeO_2_ and MnO_X_/HY catalysts, respectively, under varying total flow rate conditions during NOCM at 700 °C. In both catalysts, C_2_H_4_ consistently emerged as the dominant product, followed by C_2_H_6_ and smaller amounts of C_2_H_2_. For MnO_X_/CeO_2_ (Figure 12), increasing the total flow rate led to a noticeable enhancement in C_2_H_4_ selectivity, rising from 58.49% at the baseline flow rate to 63.22% at a 25% higher flow rate. C_2_H_6_ selectivity also increased slightly, while C_2_H_2_ selectivity decreased, indicating that higher flow rates help suppress secondary pathways responsible for acetylene formation.^46,47^

**Figure 14:**
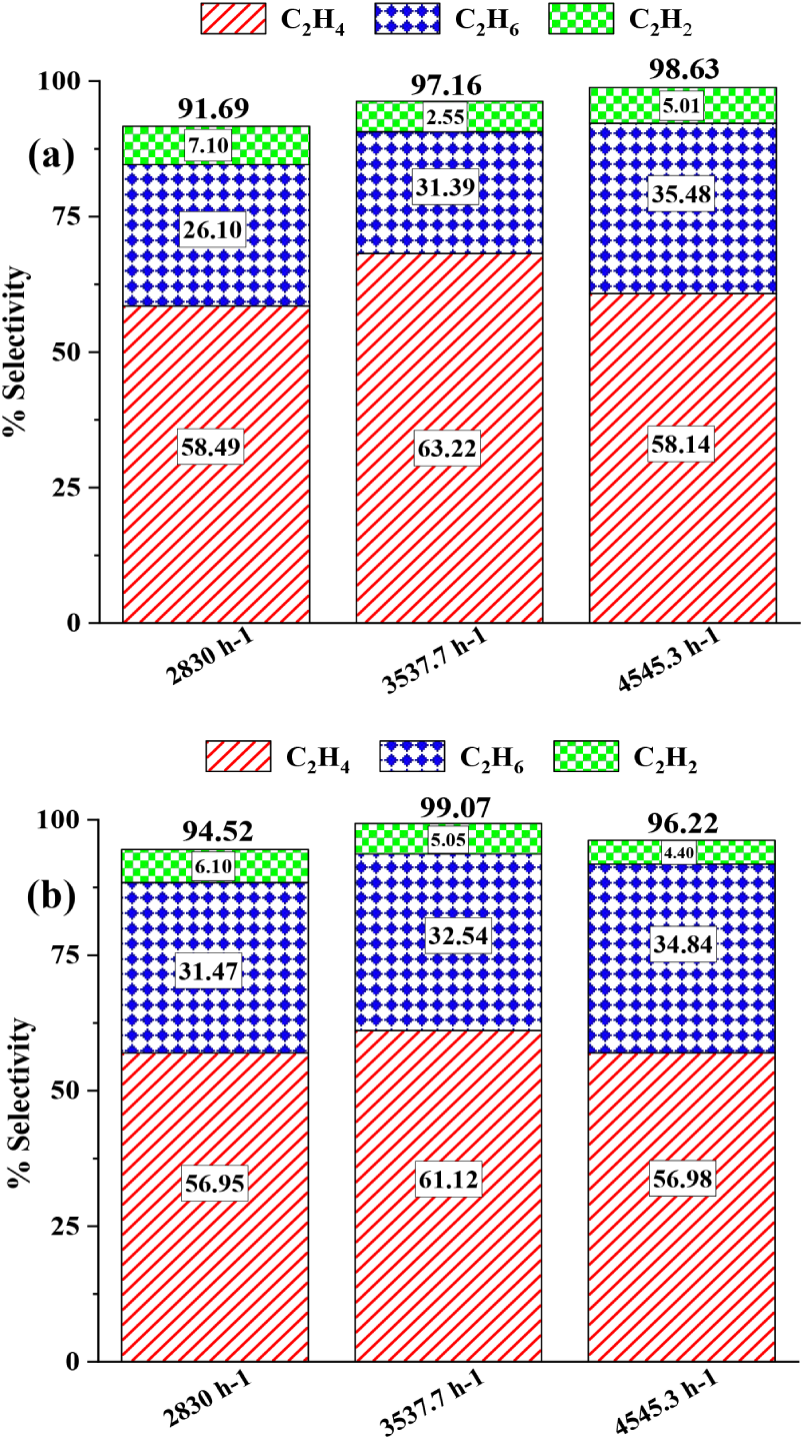
Product distribution under varying flow rate conditions for 5 wt % MnO_X_ incorporated (a) CeO_2_ and (b) HY-supported catalysts during NOCM at 700 °C. The effect of flow rate on C_2_H_4_, C_2_H_6_, and C_2_H_2_ selectivity is shown for each catalyst system.

A similar pattern was observed for the MnO_X_/HY catalyst (Figure 13), where C_2_H_4_ selectivity improved from 56.95% to 61.12% with increasing flow rate, while the proportion of C_2_H_2_ was reduced. This shift in product distribution can be attributed to the shortened residence time at higher flow rates, which reduces the likelihood of secondary hydrogen abstraction and undesired polymerization reactions. The decrease in C_2_H_2_ formation suggests that at elevated flow rates, reactive intermediates such as CH_X_ and C_2_H_X_ species are more effectively stabilized, promoting the formation of more desirable C_2_ olefins.^47^

These trends emphasize that optimizing flow rate is critical for maximizing C_2_H_4_ production while minimizing byproducts. Higher flow rates improve C_2_ selectivity by reducing secondary reactions and limiting further conversion of C_2_H_4_ into heavier species or coke precursors. However, excessively high flow rates may also lower CH_4_ conversion, due to reduced catalyst-reactant contact time. The superior performance of Mn-promoted catalysts under these varying conditions can be linked to their enhanced redox properties and surface acidity, as confirmed by H_2_-TPR and NH_3_-TPD results. In addition, BET surface area analysis indicated that the textural properties of the catalysts, particularly surface area and porosity, play a key role in providing sufficient active sites and improving accessibility, thereby supporting the observed enhancement in C_2_H_4_ selectivity.

These findings highlight that careful tuning of both catalyst properties and reaction parameters, such as flow rate, can significantly enhance the efficiency of microwave-assisted NOCM for C_2_H_4_ production. Moreover, the ability of MnO_X_/CeO_2_ to sustain high C_2_H_4_ selectivity across different flow rates reflects the beneficial role of strong metal–support interactions and oxygen mobility in stabilizing reaction intermediates, offering practical insights for designing more effective NOCM processes.

#### 3.2.3 Performance Comparison between This Work and the Literature

A literature review for the methane conversion and product selectivity studies in the methane coupling studies has been presented in Table 2. As indicated below, many systems reported in the literature typically need high reaction temperatures over 900 °C to obtain a significant CH_4_ conversion level, but with relatively undesirable C_2_ selectivity and high CO_2_ and aromatics formation. On the other hand, the MnO_X_-supported catalysts prepared in this study exhibited significantly improved performance, achieving CH_4_ conversions of 15% with MnO_X_/CeO_2_ and 12% with MnO_X_/HY with exceptionally high C_2_ selectivity of 97.16% and 99.07%, respectively. In comparison, CO_2_ and aromatic formation remained below 0.3% across both systems. These results highlight the potential of MnO_X_-based catalysts for achieving efficient and selective CH_4_ coupling at relatively lower temperatures, demonstrating their potential for further development in NOCM processes.

**Table 2:** Comparison of the Performance of Different Catalytic Systems under NOCM Conditions

					**% Product Selectivity**				
**Reaction**	**Temp (°C)**	**Catalyst**	**Feed Compositio**n	**%CH**_**4**_ **Conversion**	**C**_**2**_	**CO**_**2**_	**Aromatics**	**Method**	**Refs**
OCM	800	Metal oxides/alpha-Al_2_O_3_	CH_4_/O_2_ 50:50	11	60	6	N/A	Fixed Bed Quartz/Stainless	^1^
NOCM	1090	Fe©SiO_2_	100% CH_4_	48.1	48.4	-	Negligible	Fixed Bed Quartz	^15^
OCM via dielectric heating	400–1000	La_2_O_3_/Al_2_O_3_, CeO_2_/Al_2_O_3_	CH_4_/O_2_ 75:25	40	Yield ∼ 3.3	High at low temperature	Low at high temperature	Packed Bed Microwave and Conventional	^48^
OCM	500–800	Li/MgO and BaBiO_3‑x_	CH_4_/O_2_/He 13.33–6.7–80 vol %	Li/MgO = 30 BaBiO_3‑x_ = 15	60	N/A	N/A	Fixed Bed Microwave	^22^
NOCOM	∼1000	Ni	CH_4_/He 25:75	6.1	83	10	10	Microwave	^49^
		Fe		10	68	35	12		
		AC		31.2	72	0	28		
NOCOM	∼1250	Ni/Fe powder AC	CH_4_/He - 25:75	45	85	5	33	Microwave	^20^
NOCM	550	H-(Fe)-ZSM-5	CH_4_/N_2_ - 50:50	40	50	N/A	N/A	Fixed Bed Microwave	^18^
NOCM	700	SiC monolith 4%Mo/H-ZSM5	CH_4_/N_2_ - 75:25	11.7	35	N/A	28	Microwave	^46^
NOCM	700	1Cs-3Mo/CeO_2_	CH_4_/N_2_ – 83–17	22	90	N/A	4	Microwave	^47^
NOCM	700	5%MnO/CeO_2_	CH_4_/N_2_ −80–20	15	97.16	<0.3	<0.2	Microwave	Current work
		5%MnO/HY		12	99.07	<0.1	<0.1		

## 4 Conclusions

NOCM was investigated over MnO_X_-supported CeO_2_ and HY (SAR = 5.1) catalysts under microwave irradiation at 700 °C. The catalytic performance was evaluated in terms of CH_4_ conversion and selectivity toward C_2_H_4_ and C_2_ hydrocarbons. Both catalysts exhibited superior activity, achieving up to 15% CH_4_ conversion, 99% overall C_2_ selectivity, and 64% C_2_H_4_ selectivity, which surpasses the values reported in the literature at comparable or higher reaction temperatures. Characterization via H_2_-TPR and in situ Raman spectroscopy revealed that Mn incorporation significantly enhanced the redox behavior and surface oxygen mobility, promoting CH_4_ activation and stabilizing intermediate oxidation states. Raman analysis further confirmed the evolution of Mn species and the suppression of coke formation under microwave-assisted reaction conditions. The localized and selective heating facilitated by microwave irradiation has enhanced C–H bond cleavage while minimizing energy losses. These findings highlight the potential of Mn-based catalysts for energy-efficient and selective CH_4_ coupling to C_2_ hydrocarbons. Future studies should explore the reaction kinetics and long-term stability of Mn-based systems under continuous microwave operation to support scale-up for industrial applications.

## Supplementary Material



## Notes

Figure S1: isotherm and pore size distribution plot of (a) MnO_X_/CeO_2_ and (b) MnO_X_/HY; Figure S2: DSC plot of MnO_X_/CeO_2_; Figure S3: DSC plot of MnO_X_/HY; Figure S4. TGA and DTG plots of (a) MnO_X_/CeO_2_ and (b) MnO_X_/HY; and Figure S5: % CH_4_ conversion achieved using (a)­MnO_X_/CeO_2_ and (b) MnO_X_/HY at 700°C temperature under varied flowrates (PDF)
