# Supplementary material for: Microwave-Driven Nonoxidative and Selective Conversion of Methane to Ethylene over Mn-Based Catalysts
Source: Ind Eng Chem Res. 2025 Nov 11;64(46):22102–14. doi: 10.1021/acs.iecr.5c02894 (PMC12636017; doi:10.1021/acs.iecr.5c02894)
Supplement: Supplementary file 1 [file ie5c02894_si_001.pdf]

Supporting Information

Microwave-driven Non-oxidative and Selective Conversion of Methane to Ethylene over Mn-based Catalysts

Snehitha Reddy Baddam, Changle Jiang, Manohar Reddy Poreddy, Kshitij Tewari, Brandon Robinson, Yuxin Wang, Srinivas Palanki, Jianli Hu*

Department of Chemical and Biomedical Engineering,

West Virginia University, Morgantown, West Virginia, United States

*Corresponding Author: Jianli Hu, john.hu@mail.wvu.edu

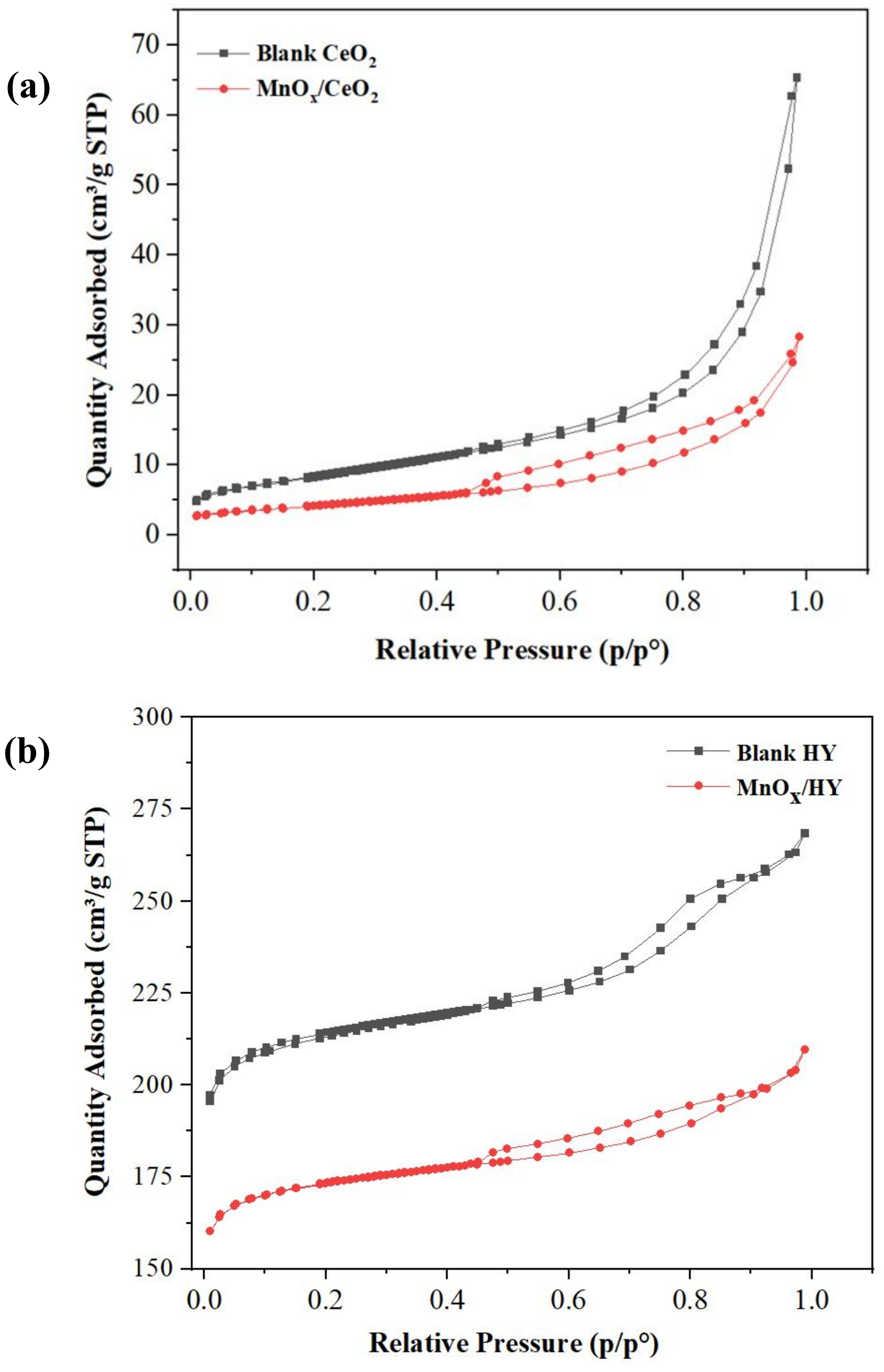

Figure S1: Isotherm and pore size distribution plot of (a) MnO_X/CeO_2 and (b) MnO_X/HY

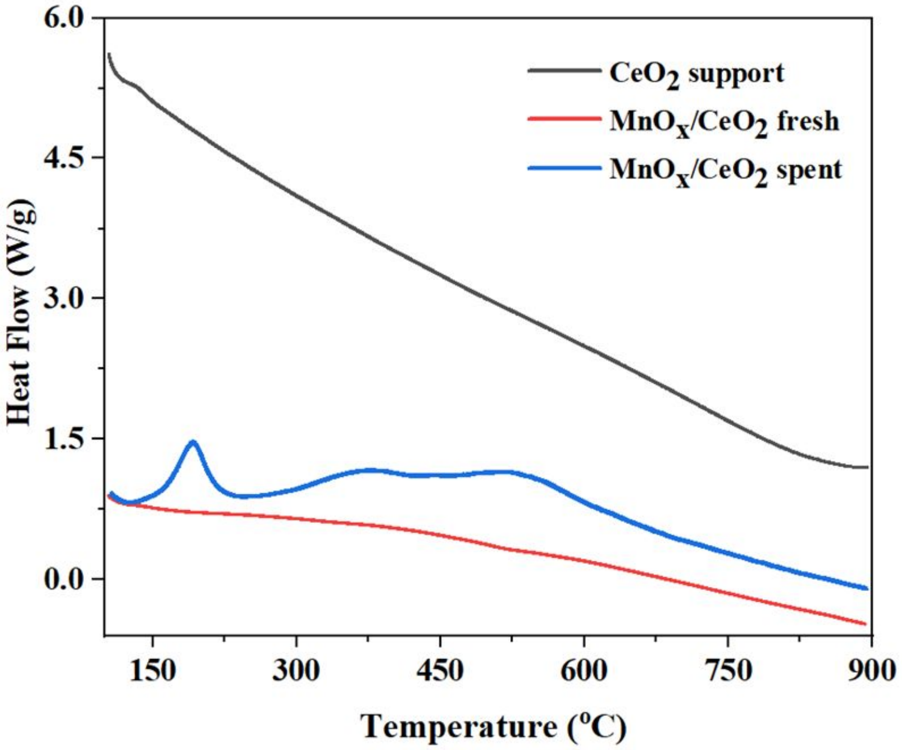

Figure S2: DSC plot of MnO_X/CeO_2

The DSC analysis confirms the TGA results by highlighting the reoxidation behavior of Mn on CeO_2. The spent MnO_X/CeO_2 catalyst exhibits a pronounced exotherm at ~150-200 °C, attributed to the reoxidation of reduced Mn species (Mn^0/MnO/MnC_X) and partial combustion of surface carbon, in agreement with the observed weight gain in TGA. A broader exothermic feature between 450-600 °C corresponds to further oxidation to higher-valence Mn oxides and oxidation of more refractory carbon species. In contrast, bare CeO_2 displays no distinct exothermic peaks, confirming its role as an oxygen buffer (Ce^{3+}/Ce^{4+}), which facilitates low-temperature Mn reoxidation.

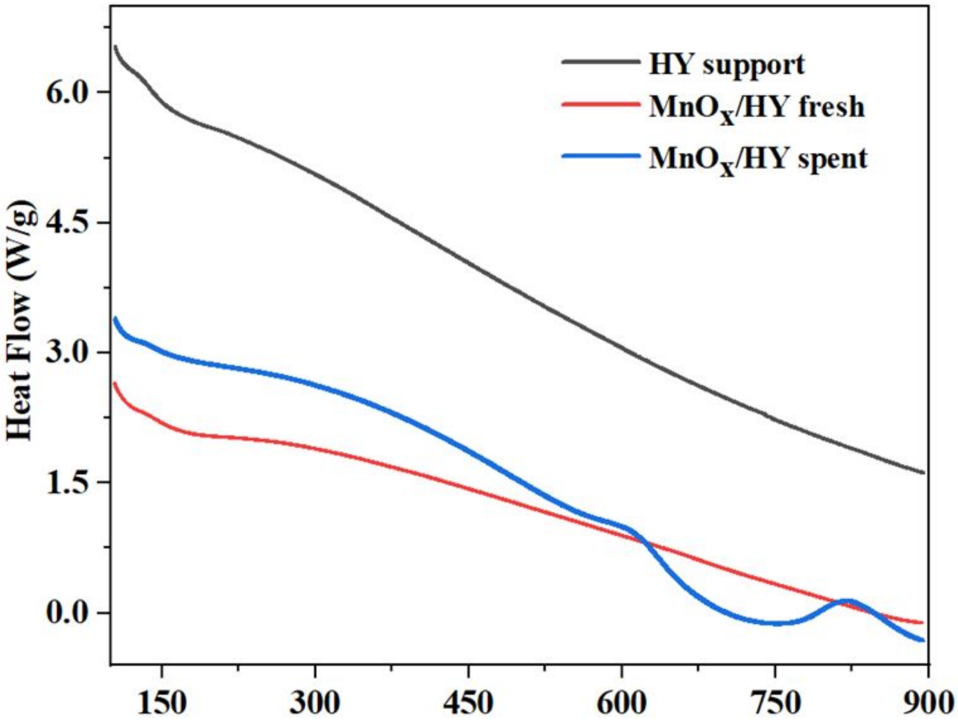

Figure S3: DSC plot of MnO_X/HY

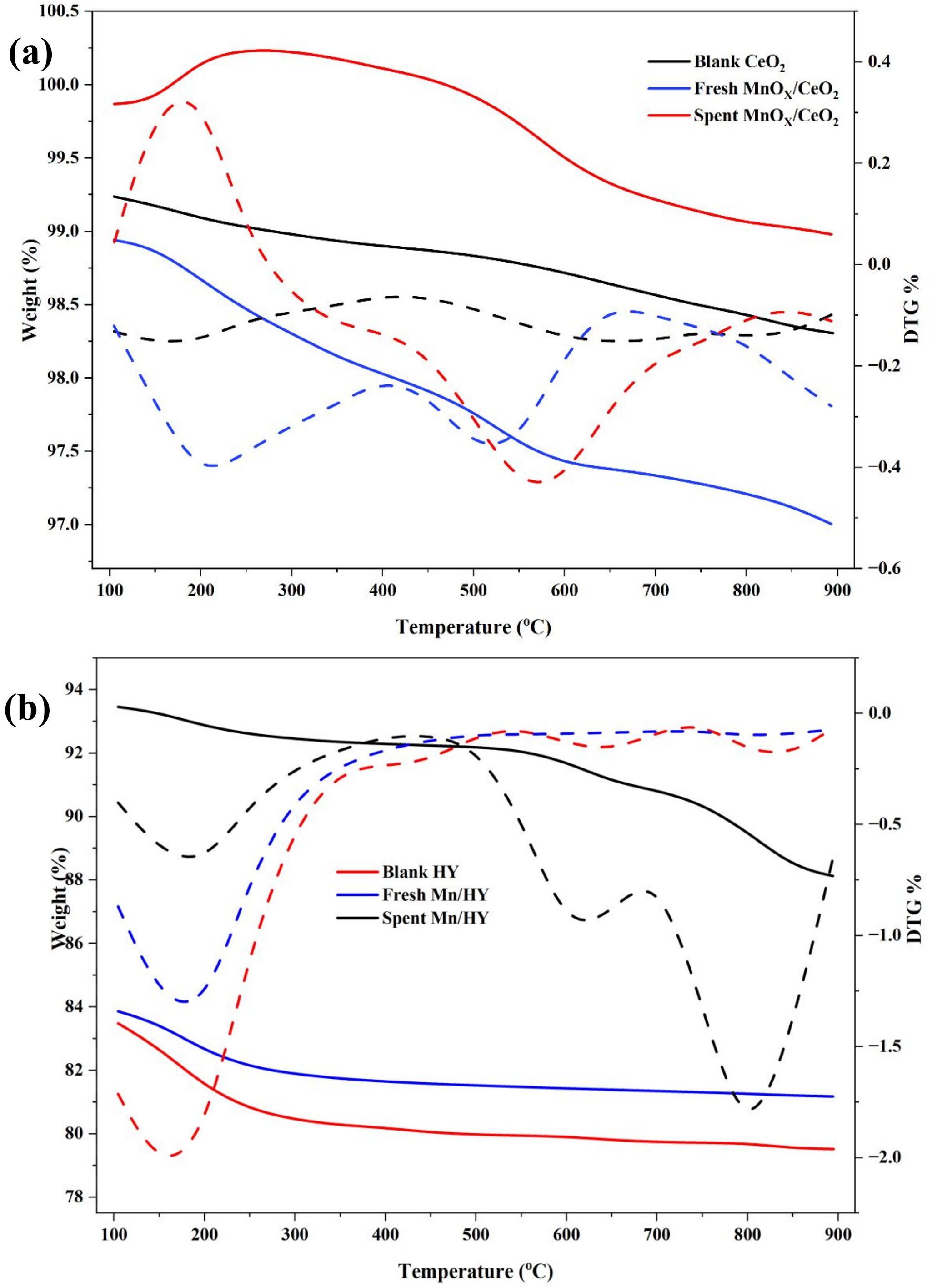

Figure S4. TGA and DTG plots of (a) MnO_X/CeO_2 and (b) MnO_X/HY

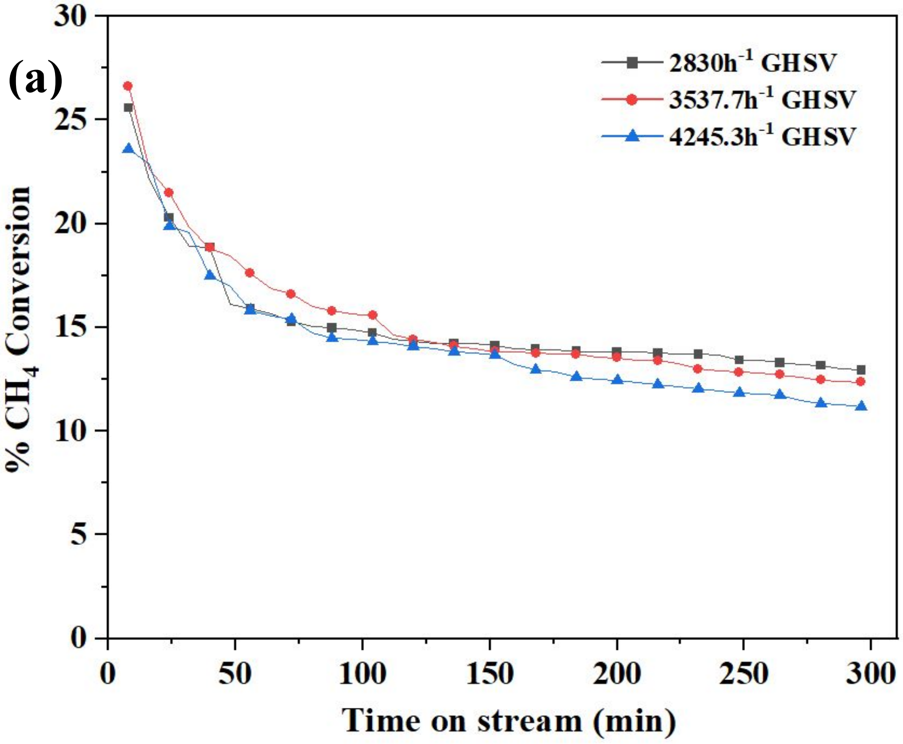

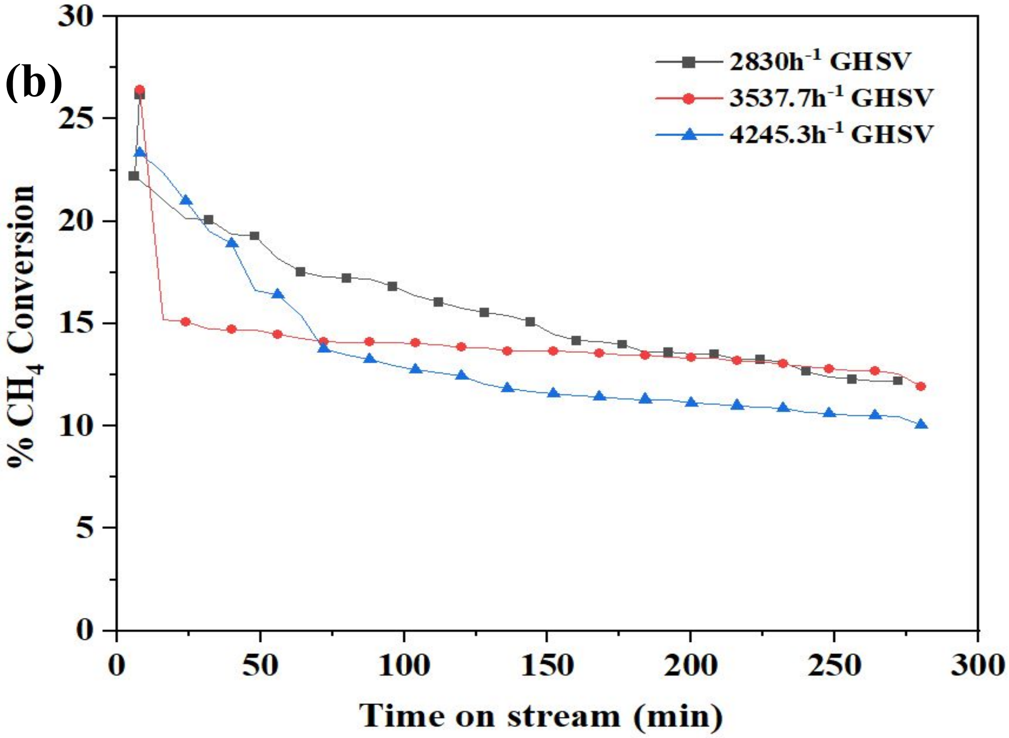

Figure S5. % CH_4 Conversion achieved using (a)MnO_X/CeO_2 and (b) MnO_X/HY at 700°C temperature under varied flowrates
